# EMBO Conference Series on the Assembly and Function of Neuronal Circuits

**DOI:** 10.1186/2042-1001-1-15

**Published:** 2011-10-27

**Authors:** Alice Y Wang, Jeremiah Y Cohen

**Affiliations:** 1 Department of Molecular and Cellular Biology, Harvard University, 16 Divinity Avenue, Cambridge, MA 02138, USA

**Keywords:** neuronal circuit function, development behavior

## Abstract

The 2011 EMBO Conference Series on the Assembly and Function of Neuronal Circuits was held from 25 to 30 September 2011 at Monte Verità in Ascona, Switzerland. Approximately 100 participants explored current challenges and approaches to studying neural circuit function and organization.

## 

The 2011 EMBO Conference Series on the Assembly and Function of Neuronal Circuits was held from 25 to 30 September 2011 at Monte Verità in Ascona, Switzerland. Approximately 100 participants explored current challenges and approaches to studying neural circuit function and organization. The conference was organized into two sessions of oral presentations per day followed by an evening lecture and a nightly poster session at the Centro Stefano Franscini. The generously timed meals and coffee breaks offered additional opportunities for more informal and intimate discussions throughout the meeting.

The topics spanned themes such as the importance of inhibition and its interplay with excitation in shaping circuit activity, the effects of neuromodulators on neurons and behavior, the genetic underpinnings of behavior and how specific neuron types wire and communicate. There was a notable trend toward the use of combinations of genetic, optical, electrophysiological and behavioral methods. We noticed a surge in the use of optogenetics for fine manipulation of genetically defined neurons, as well as many studies in awake, behaving animals, with the use of imaging or electrophysiology. A wide range of model organisms was used, from the traditionally popular *Caenorhabditis elegans*, *Drosophila*, zebrafish, songbirds, rodents and macaques to electric fish, turtles and Egyptian fruit bats. In addition to experimental approaches, a few computational studies were presented that provide models to describe circuit dynamics, such as the influence of cortical inhibition.

Herein we sample a few highlights from the meeting that showcase the breadth of current research in neuronal circuits. Owing to space limitation, we apologize that we cannot comment on all of the presentations.

## The role of inhibition in shaping cortical circuits

Massimo Scanziani presented work on the mouse primary visual cortex (V1) and lateral geniculate nucleus (LGN). He expressed light-activated opsins, Channelrhodopsin-2 (ChR2) and archaerhodopsin (Arch) in layer VI (L6) pyramidal neurons and found that L6 excitation decreases firing rates in more superficial layers (presumably via interneurons) and in LGN (presumably via the thalamic reticular nucleus). Sonja Hofer, who received a prize for the best short oral presentation, used a combination of two-photon microscopy and patch-clamp recordings to study the connectivity and tuning preferences of different cell types in mouse V1. She found that whereas excitatory pyramidal neurons receive sparse input from neurons with similar tuning preferences, parvalbumin (PV)-positive GABAergic interneurons receive input with high probability from neurons with diverse stimulus preferences. Yang Dan expressed ChR2 in subsets of interneurons and Arch in pyramidal neurons in mouse V1. Exciting PV neurons sharpened orientation tuning and enhanced direction selectivity (and also appeared to have an effect on the mouse's ability to discriminate stimuli of different orientations). Exciting somatostatin neurons enhanced direction selectivity but did not change orientation tuning. Kevin Franks examined how recurrent connectivity in the piriform cortex (PCx) gates sensory information from the olfactory bulb. He found that a distributed and excitatory recurrent network in PCx activates strong feedback inhibition such that, if recurrent activity precedes sensory input, feedback inhibition will arise to suppress bulb-evoked spiking.

## Neuromodulators

Barry Dickson presented his work on how neural circuits in the male *Drosophila *brain learn to select for sexually receptive females. He found that courtship learning depends on the activation of a specific class of dopaminergic neurons in the protocerebrum which project to neurons with a subtype of dopamine receptors in the mushroom body. David Anderson presented new techniques for the study of the role of dopaminergic neurons involved in hunger in *Drosophila*.

Cori Bargmann probed the genetic mechanisms that allow *C. elegans *to decide whether to exploit a food patch or to abandon it and explore other options. She found that this decision depends on the expression of the catecholamine receptor tyra-3, a homolog of the vertebrate adrenergic receptor. Alex Shier described the importance of hypocretin in sleep regulation in larval zebrafish and showed a promising technique for identifying drug targets using high-throughput behavioral screening.

## Dendritic computation

Dendritic computation was featured in several studies. Jackie Schiller demonstrated supralinear summation between thalamocortical and corticocortical synapses via *N*-methyl-D-aspartate receptors at cortical dendrites. Bartlett Mel described theoretical approaches to understanding excitatory and inhibitory synaptic input to dendrites and stressed that dendrites must be considered processing units in their own right. Michael Haüsser showed *in vivo *patch recordings from orientation-tuned mouse V1 dendrites. Erin Schuman showed the existence of many mRNAs at dendrites, suggesting a place to look for a molecular basis for dendritic computation.

## Development

A few talks addressed how neurons find their targets during development. Andrew Huberman described work addressing the problem of how retinal ganglion cells find their targets in the brain. Liqun Luo showed how the *Drosophila *olfactory system wires, and Oscar Marín showed how Cajal-Retzius cells in layer I in cortex are distributed across the future cortical surface. Tania Rinaldi Barkat showed that the auditory critical period for thalamocortical modification depends on telencephalin, a membrane adhesion protein, and that its loss induces precocious spine maturation on pyramidal cells in layer IV of the primary auditory cortex.

## Neuronal activity and behavior

Nirao Shah showed that a sexually dimorphic gene with higher expression in the male mouse bed nucleus of the stria terminalis and medial amygdala may play a role in sexual behavior. Allison Doupe and Bence Ölveczky presented recordings from nuclei in songbirds to demonstrate the role of variability in producing learned motor patterns. Peer Wulff presented a study on how the selective ablation of PV neurons in the medial prefrontal cortex of mice impairs spatial working memory and cognitive flexibility. Daniel Salzman showed that neurons in the monkey basolateral amygdala (BLA) may contribute to selective attention via their projections to extrastriate cortex. Single BLA neurons showed sustained excitation or inhibition from stimuli that indicate high- or low value rewards. Adi Mizrahi showed that, by patching neurons in primary auditory cortex, pup odors modulate responses to ultrasonic pup vocalizations in lactating mothers, but not in naïve virgins. Alla Karpova observed abrupt and synchronous firing of neuronal ensembles in medial prefrontal cortex during transitions in behavioral strategies of rats performing two-alternative choice tasks. Nachum Ulanovsky demonstrated a large-scale cognitive map in bats and presented recordings from place cells in the hippocampus. He showed three-dimensional representations of space in the hippocampus as bats flew in the laboratory.

## Building a connectome

Jeff Lichtman showed how axons at the neuromuscular junction connect to the muscle and showed preliminary efforts to image the mouse cortical connectome via large-scale serial electron microscopic reconstruction. Hongkui Zheng, from the Allen Institute for Brain Science, presented new initiatives to provide a whole-brain, three-dimensional map of axonal projections using cell-type specific creatinine (Cre) lines and Cre-dependent viral tracers.

Amid talks about the remarkable wiring specificity of particular circuits, Haim Sompolinsky gave a thought-provoking lecture on how random connectivity may be an efficient strategy for encoding of sensory stimuli. He reviewed new developments in the field of compressed sensing and offered a glimpse of its potential use in neuroscience.

The diversity of topics covered at this year's conference reflects the wide range of approaches neuroscientists currently use to study neuronal circuits. From examining the molecules and genes of specific cell-types to developing models of how networks of randomly connected neurons behave, the study of neuronal circuits is rapidly expanding, and we predict that it will continue to grow in the future.

## Abbreviations

Arch: archaerhodopsin; BLA: basolateral amygdala; ChR2: channelrhodopsin-2; LGN: lateral geniculate nucleus; PCx: piriform cortex; PV: parvalbumin; V1: primary visual cortex.

## Competing interests

The authors declare that they have no competing interests.

## Authors' contributions

AYW and JYC wrote the meeting report, and both authors approved the final manuscript.

